# Production of (*R*)-3-Quinuclidinol by *E. coli* Biocatalysts Possessing NADH-Dependent 3-Quinuclidinone Reductase (QNR or bacC) from *Microbacterium luteolum* and *Leifsonia* Alcohol Dehydrogenase (LSADH)

**DOI:** 10.3390/ijms131013542

**Published:** 2012-10-19

**Authors:** Kentaro Isotani, Junji Kurokawa, Nobuya Itoh

**Affiliations:** Department of Biotechnology, Faculty of Engineering, Biotechnology Research Center, Toyama Prefectural University, 5180 Kurokawa, Imizu, Toyama 939-0398, Japan; E-Mails: s976001@st.pu-toyama.ac.jp (K.I.); z16072@st.pu-toyama.ac.jp (J.K.)

**Keywords:** 3-quinuclidinone reductase (QNR and bacC), *Microbacterium luteolum* JCM 9174, *Leifsonia* alcohol dehydrogenase (LSADH), (*R*)-(−)-3-quinuclidinol, optically pure alcohol, immobilized biocatalyst

## Abstract

We found two NADH-dependent reductases (QNR and bacC) in *Microbacterium luteolum* JCM 9174 (*M. luteolum* JCM 9174) that can reduce 3-quinuclidinone to optically pure (*R*)-(−)-3-quinuclidinol. Alcohol dehydrogenase from *Leifsonia* sp. (LSADH) was combined with these reductases to regenerate NAD^+^ to NADH *in situ* in the presence of 2-propanol as a hydrogen donor. The reductase and LSADH genes were efficiently expressed in *E. coli* cells. A number of constructed *E. coli* biocatalysts (intact or immobilized) were applied to the resting cell reaction and optimized. Under the optimized conditions, (*R*)-(−)-3-quinuclidinol was synthesized from 3-quinuclidinone (15% *w*/*v*, 939 mM) giving a conversion yield of 100% for immobilized QNR. The optical purity of the (*R*)-(−)-3-quinuclidinol produced by the enzymatic reactions was >99.9%. Thus, *E. coli* biocatalysis should be useful for the practical production of the pharmaceutically important intermediate, (*R*)-(−)-3-quinuclidinol.

## 1. Introduction

The chemical processes for obtaining optically pure compounds include enantiomer separation from a racemic mixture, derivation from natural substances and asymmetric synthesis. Of these, asymmetric organic synthesis is the most efficient and useful method of producing chiral synthons. Chiral metal catalysts such as BINAP-Ru [1] and chiral Co (II) salen complex [2,3] have been successfully used as chemical catalysts for synthesizing chiral alcohols or chiral diols from various ketones or epoxides in a number of cases. However, trace metal contamination left in the products and the high cost of catalysts are unresolved difficulties affecting many reactions. To overcome the issues of conventional processes, biocatalytic transformation using enzymes or microorganisms has been applied to the asymmetric reduction of ketones. These biocatalytic processes are more environmentally sustainable and thus more attractive for pharmaceutical manufacturing [4]. In recent years, the asymmetric reduction of ketones with biocatalysts has been reported for the production of chiral alcohols and applied to industry [5–9].

(*R*)-(−)-3-Quinuclidinol, which has a bicyclic structure with a bridgehead nitrogen, is a valuable intermediate for pharmaceuticals. It has been used as the chiral synthon for a cognition enhancer, a bronchodilator and a urinary incontinence agent [10]. The optical resolution of (**±**)-3-quinuclidinol esters by the hydrolysis reaction of protease has been reported [11]. Moreover, a number of enzymes have been reported as catalysts for the reduction of 3-quinuclidinone to (*R*)-(−)-3-quinuclidinol [12–14]. To accelerate the bioreduction process, it is necessary to regenerate NAD(P)^+^ to NAD(P)H *in situ*. There have been many reports on the reproduction of NAD(P)H via coupling reactions using formate/formate dehydrogenase (FDH) [15] and glucose/glucose dehydrogenase (GDH) [16]. In the regeneration of NAD(P)H, 2-propanol is another suitable hydrogen donor because of its chemical properties and low cost [17,18]. Recently, our research group reported an excellent alcohol dehydrogenase from *Leifsonia* sp. strain S749 (LSADH) for the synthesis of chiral alcohols and concomitant regeneration of NADH with 2-propanol [19–21]. Biocatalytic reaction using whole cells is more stable than isolated enzyme reaction, and can reduce the cost of catalysts and cofactors [21,22].

**Figure 1:**
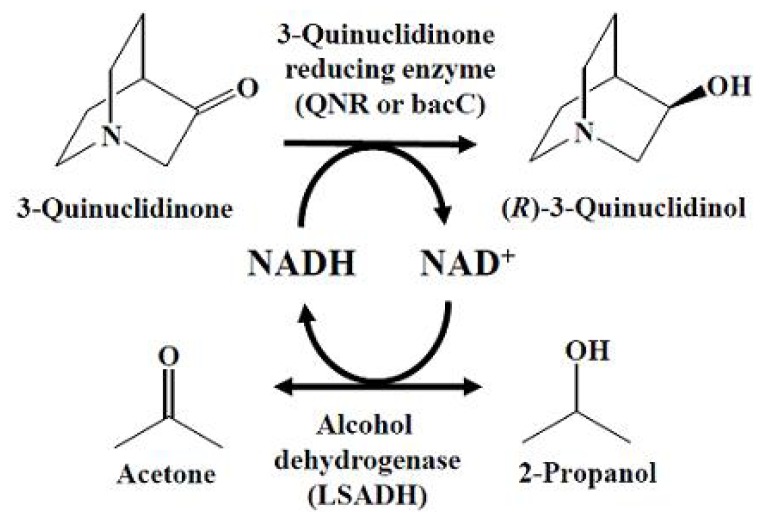
(*R*)-(−)-3-Quinuclidinol production system with 3-quinuclidinone reductase (QNR) and *Leifsonia* alcohol dehydrogenase (LSADH).

In this study, we report a bioreduction system for synthesizing (*R*)-(−)-3-quinuclidinol using a recombinant *E. coli* cell biocatalyst possessing 3-quinuclidinone reductase (QNR or bacC) from *M. luteolum* JCM 9174 and LSADH (Figure 1). The *bacC* gene, which consists of 768 nucleotides corresponding to 255 amino acid residues and is a constituent of the bacilysin synthetic gene cluster, was obtained by PCR based on homology with known genes [23]. The *qnr* gene consisted of 759 nucleotides corresponding to 252 amino acid residues [24]. Both enzymes belong to the short-chain alcohol dehydrogenase/reductase (SDR) family. QNR showed 37% homology with the bacC isolated from *M. luteolum*. The resulting *E. coli* biocatalyst exhibited a high level of production of (*R*)-(−)-3-quinuclidinol and enantioselectivity (150 mg/mL, >99.9% e.e.).

## 2. Results and Discussion

### 2.1. Construction of Expression Vector of QNR and LSADH

pET28a (Merck KGaA, Darmstadt, Germany) and pRSFDuet-1 (Merck) vectors have the kanamycin resistance gene. Therefore, it was not suitable to select the colony having both plasmids. Thus, the *qnr* gene was subcloned into *Nde*I-*Hind*III sites of pETDuet-1, which has an ampicillin resistance gene, from pET28-QNR [24] to give pETDuet-QNR. The *lsadh* gene was amplified by PCR with the following primers and using pKELA [20] as a template: LSADHforNde (5′-GAGATCATATGGCTCAGTACGACGTC-3′) (the *Nde*I site is underlined) and LSADHrevSal (5′-TTTGTCGACTCACTGGGCGGTGTAG-3′) (the *Sal*I site is underlined) under the following conditions: 94 °C for 2 min, followed by 98 °C for 10 s, 60 °C for 30 s and 68 °C for 1 min for a total of 30 cycles in accordance with the manufacturer’s protocol for KOD FX DNA polymerase (Toyobo, Osaka, Japan). The PCR fragments were digested with *Nde*I and *Sal*I and inserted into *Nde*I and *Xho*I sites of pRSFDuet-1 (Merck) to obtain pRSFDuet-LSADH. For the co-expression of the *qnr* and *lsadh*, *E. coli* BL21 (DE3) was transformed with pETDuet-QNR and pRSFDuet-LSADH. Unfortunately, we could not construct pETDuet-QNR-LSADH.

### 2.2. Construction of Expression Vector of bacC and LSADH

**Figure 2:**
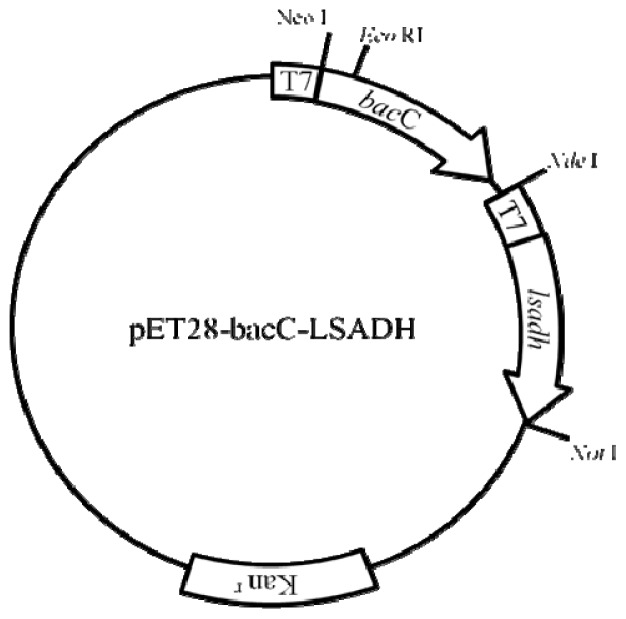
pET28-bacC-LSADH plasmid.

The PCR fragment of *lsadh* mentioned above was digested with *Nde*I and *Sal*I, and then cloned into the *Nde*I and *Xho*I sites of pETDuet-1 to obtain pETDuet-LSADH. Then, to add the T7 promoter region into the upstream of the *lsadh* gene, the fragment was amplified again by PCR with the pETDuet-LSADH as a template and the following primers: pETUpstream-69214-3 (5′-ATGCGTCCGGCGTAGA-3′) and LSADHrevSal. The amplicon was digested with *Hin*dIII and *Sal*I, and cloned into *Hin*dIII and *Xho*I sites of the pET28-bacC [23] to generate pET28-bacC-LSADH, in which the *bacC* and *lsadh* genes were connected in this order and each gene was under the control of the T7 promoter (Figure 2).

### 2.3. Enzymatic Activity of the *E. coli* Biocatalyst

Table 1 shows the results of the enzymatic activity of each *E. coli* biocatalyst. The activities of *E. coli* BL21(DE3)/pET28-QNR, *E. coli* BL21(DE3)/pET28-bacC and *E. coli* BL21/pKELA, in which the gene was independently expressed, indicated higher 3-quinuclidinone-reducing/2-propanol oxidizing activity than *E. coli* BL21 (DE3)/pETDuet-QNR/pRSFDuet-LSADH and *E. coli* BL21 (DE3)/pET28-bacC-LSADH, in which the two genes were co-expressed. The results suggested that the combination of two *E. coli* biocatalysts, for example, *E. coli* BL21(DE3)/pET28-QNR and *E. coli* BL21/pKELA, should be suitable for the conversion of 3-quinuclidinone.

Moreover, in order to increase the durability of the biocatalyst, we immobilized the recombinant *E. coli* cells including *E. coli* BL21(DE3)/pET28-QNR, *E. coli* BL21(DE3)/pET28-bacC and *E. coli* BL21/pKELA by coating the cell surface with polyethyleneimine (PI) and glutaraldehyde (GA) [25]. Unfortunately, the activity of immobilized biocatalyst was not determined because the enzyme could not be extracted from the immobilized cells.

### 2.4. Evaluation of *E. coli* Biocatalyst and Optimization of the Reaction

Based on the enzyme activity shown in Table 1, we applied various combinations of biocatalysts to (*R*)-(−)-3-quinuclidinol synthesis. Table 2 summarizes the production level of (*R*)-(−)-3-quinuclidinol and the molar conversion yield. In all cases, the absolute configuration of (*R*)-(−)-3-quinuclidinol produced was >99.9%. Conversions of almost 100% were attained except for the combination of *E. coli*/pET28-bacC and *E. coli*/pKELA (ratio: 1:4), when the final 10% (*w*/*v*, 626 mM) of 3-quinuclidinone was employed in the reaction mixture. Low production was due to the insufficient 3-quinuclidinone-reducing activity of this combination of biocatalysts. Neither QNR nor bacC catalyzes the reverse reaction, (*R*)-(−)-3-quinuclidinol oxidation to 3-quinuclidinon [23,24]; therefore, the unfavorable equilibrium between alcohol and ketone was negligible. However, it was noted that the conversion yield decreased when 15% (*w*/*v*, 939 mM) 3-quinuclidinone was employed in the reaction mixture, even if twice the amount of biocatalyst was added. During the course of the study, we observed that more than 5% of 3-quinuclidinone in the reaction mixture seemed to inhibit the reaction rate, suggesting that a high concentration of 3-quinuclidinone has an inhibitory effect on the enzyme catalysts. Thus, we adopted a method of consecutive additions of 3-quinuclidinone (5% *w*/*v*) and 2-propanol (5% *v*/*v*) to the reaction mixture at 8 h intervals. Under the optimized conditions, combinations of *E. coli*/pET28-QNR and *E. coli*/pKELA (4:1), *E. coli*/pET28-QNR and *E. coli*/pKELA (1:1) and *E. coli*/pETDuet-QNR/pRSFDuet-LSADH biocatalysts gave complete conversions of 10% 3-quinuclidinone to (*R*)-(−)-3-quinuclidinol.

**Figure 3:**
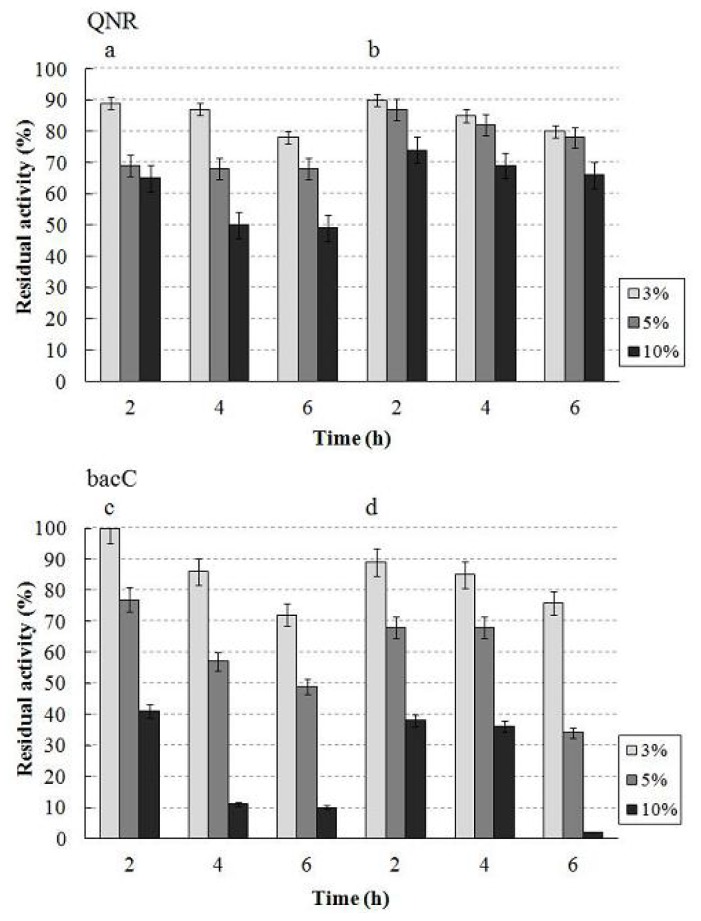
Effect of organic solvents on enzyme stability. Residual activity is shown after incubation of designated concentration of 2-propanol or acetone and time for the purified enzymes. (**a**) 2-propanol on QNR; (**b**) acetone on QNR; (**c**) 2-propanol on bacC; (**d**) acetone on bacC. Data are the mean value of three independent measurements with standard deviation as shown.

**Figure 4:**
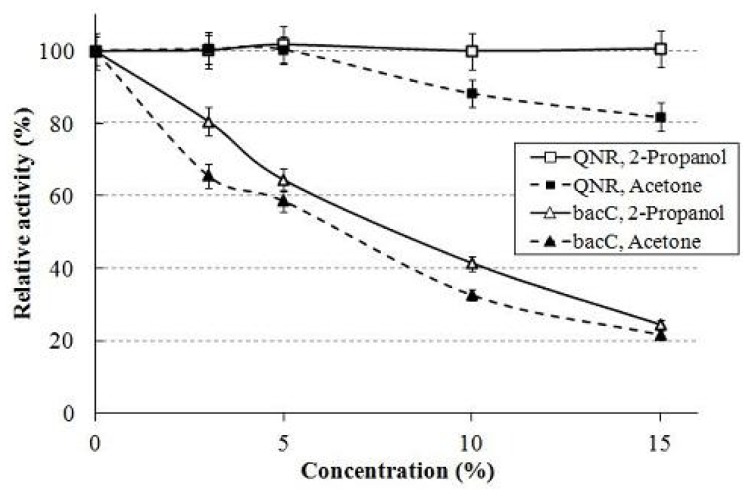
Effect of organic solvents on enzyme activity. Activity of QNR and bacC was measured in the presence of 0, 3, 5, 10 and 15% (*v*/*v*) 2-propanol/acetone in the assay mixture. Enzyme activity in the presence of 2-propanol is shown as open squares for QNR and open triangles for bacC. Activity in the presence of acetone is shown as closed squares for QNR and closed triangles for bacC. Data are the mean value of three independent measurements with standard deviation as shown.

We measured the activity and stability of purified QNR and bacC [23,24] at various concentrations to check the effects on the enzymes of 2-propanol and the acetone produced. Polar organic solvents such as 2-propanol and acetone are known to inhibit enzyme activity [25]. QNR showed greater stability than bacC in 2-propanol-KPB (pH 7.0) or acetone-KPB (pH 7.0) medium (Figure 3). BacC was especially unstable in both 10% mediums. Moreover, the specific activity of bacC in 2-propanol-KPB (pH 7.0) or acetone-KPB (pH 7.0) medium decreased as the concentrations of 2-propanol and acetone increased, while QNR barely decreased (Figure 4). The decline in specific activity of bacC in these media was much greater than that of QNR. Thus, the data confirmed that QNR was superior to bacC for the production of (*R*)-(−)-3-quinuclidinol in 2-propanol-KPB (pH 7.0) medium. The results also suggested that it is important to keep the concentrations of 2-propanol and acetone lower than 10% during the reaction. Therefore, consecutive additions of 3-quinuclidinone (5% *w*/*v*) and 2-propanol (5% *v*/*v*) to the reaction mixture at 8 h intervals suppressed the inhibitory effect of the polar organic solvent. In addition, an open reaction system without sealing was adopted to promote the vaporization of acetone from the reaction mixture. Itoh *et al.* reported that aeration is another effective method in asymmetric bioreduction processes using LSADH with 2-propanol to reduce the concentration of acetone [21].

However, it was impossible to accumulate more than 10% of (*R*)-(−)-3-quinuclidinol product in the reaction mixture using intact *E. coli* cells biocatalysts even after consecutive additions of the substrate and 2-propanol (Table 2).

### 2.5. Evaluation of Immobilized *E. coli* Biocatalyst and the Conversion Time Course

**Figure 5:**
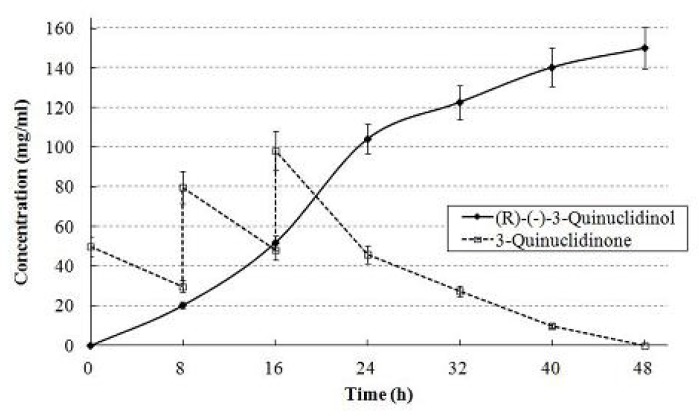
Time course of the production of (*R*)-(−)-3-quinuclidinol by immobilized *E. coli* biocatalysts. The concentration of (*R*)-(−)-3-quinuclidinol is represented by a solid line and that of 3-quinuclidinone by a dashed line. Data are the mean value of three independent measurements with standard deviation as shown.

Immobilization of recombinant *E. coli* cells often increases the operational stability of biocatalysts in the synthetic reaction. Itoh *et al.* reported a simple immobilization method for recombinant *E. coli* biocatalyst (pKELA, LSADH) using PI and GA [26], and revealed its superior operational stability for the bioreduction of 4-hydroxy-2-butanone to (*R*)-1,3-butanediol in 10% 2-propanol-KPB (pH 7.0) medium [25]. We applied this method for the immobilization of *E. coli*/pET28-QNR, *E. coli*/pET28-bacC and *E. coli*/pKELA. Complete conversion of 15% (*w*/*v*) (150 mg, 939 mM) 3-quinuclidinone to (*R*)-(−)-3-quinuclidinol was attained by a combination of immobilized *E. coli*/pET28-QNR and *E. coli*/pKELA, although the reaction time was extended (Figure 5 and Table 2). Low production of immobilized *E. coli*/pET28-bacC and *E. coli*/pKELA was probably due to the loss of bacC activity during the immobilization procedure. We speculated that the PI and GA polymer matrix constructed on the *E. coli* cell surface has cationic properties due to unreacted amino and imino groups of PI and hinders the access of cationic 3-quinuclidinone to the enzymes in the cells, making it possible to overcome the inhibitory effect of a high concentration of substrate. However, the lower accessibility of substrate to immobilized cells compared with intact cells prolonged the reaction time.

The production level of (*R*)-(−)-3-quinuclidinol in this study was the highest ever reported, indicating that *E. coli* biocatalysis would provide a practical method of producing important chiral compounds.

## 3. Experimental Section

### 3.1. Chemicals

3-Quinuclidinone hydrochloride was obtained from Sigma-Aldrich Chemical Co. (WI, USA). 3-Quinuclidinol and glutaraldehyde were purchased from Tokyo Chemical Industry Co., Ltd. (Tokyo, Japan), and (*R* )-(−)-3-quinuclidinol from Kanto Chemical Co. (Tokyo, Japan). Polyethylenimine P-70 solution (30% (*v*/*v*) solution) was purchased from Wako Pure Chemical Industries (Osaka, Japan). All other chemicals used in this study were of analytical grade and commercially available.

### 3.2. Methods

#### 3.2.1. Preparation of *E. coli* Biocatalysts Overproducing Recombinant Enzymes

*E. coli* BL21 (DE3)/pET28-QNR or *E. coli* BL21 (DE3)/pET28-bacC [23,24] were each grown in Luria-Bertani (LB) medium consisting of 1.0% (*w*/*v*) tryptone, 0.5% yeast extract and 0.5% NaCl (pH 7.0). Pre-cultivation was carried out in the medium (4 mL) containing 0.05 mg mL^−1^ kanamycin sulfate in a test tube for 12 h at 37 °C with shaking (180 rpm). One milliliter of the culture medium was added to fresh medium (100 mL) containing antifoam PE-H (final concentration of 0.1%), 0.05 mg mL^−1^ kanamycin sulfate and 0.4 mM isopropyl-β-d-1-thiogalactopyranoside (IPTG) in a shaking flask at 120 rpm and cultured at 37 °C for 24 h. After centrifugation (10,000*g*, 5 min), the precipitated cells from 100 mL of culture were washed once with 100 mM potassium phosphate buffer (KPB) (pH 7.0) and used as a biocatalyst. *E. coli* BL21/pKELA was cultured according to our previous paper [20]. Cultivation of *E. coli* BL21(DE3)/pET28-bacC-LSADH was performed in the same manner as *E. coli* BL21(DE3)/pET28-QNR and *E. coli* BL21(DE3)/pET28-bacC. *E. coli* BL21(DE3)/pETDuet-QNR/pRSFDuet-LSADH were cultured with 0.05 mg mL^−1^ kanamycin sulfate, 0.1 mg mL^−1^ ampicillin and 0.4 mM IPTG. Cultured cells (1 mL) were disrupted by a sonication device (Ultrasonic Disruptor, Tomy Seiko, Tokyo, Japan), repeated three times at 40 W for 10 s with 10 s intervals for cooling. After centrifugation (10,000*g*, 20 min), the enzymatic activity in the supernatant was assayed as described below.

#### 3.2.2. Immobilization Procedure of *E. coli* Cells

*E. coli* BL21 (DE3)/pET28-QNR, pET28-bacC and *E. coli* BL21/pKELA cells were immobilized after cultivation by the method described by Itoh *et al.* [25] with 3% (*w*/*v*) polyethyleneimine (PI) and 0.5% (*w*/*v*) glutaraldehyde (GA). These immobilized biocatalysts were also used for the reduction of 3-quinuclidinone.

#### 3.2.3. Enzyme Assay

A spectrophotometric assay of 3-quinuclidinone reductase activity was performed by measuring the decrease in absorbance of NADH at 340 nm (ɛ = 6.22 mM^−1^ cm^−1^). The assay was performed in a reaction mixture consisting of 10 μmol of substrate, 0.3 μmol of NADH, 50 μmol of KPB (pH 7.0) and 10 μL of enzyme solution in a total volume of 1.0 mL. LSADH activity with 2-propanol (50 mM) was measured in the oxidative reaction. The reaction mixture consisted of 1 μmol NAD^+^, 50 μmol 2-propanol, 100 μmol KPB (pH 7.0) and 10 μL enzyme solution in a total volume of 1.0 mL. One enzyme unit was defined as the amount of enzyme that converted 1 μmol of NADH or NAD^+^ per min at 25 °C.

#### 3.2.4. Effect of Organic Solvent for Enzymes

The stability of the purified enzymes (500 μg) [23,24] was measured after incubation in 2-propanol/acetone and 100 mM KPB (pH 7.0) medium at various concentrations in a total volume of 1 mL for 2, 4 and 6 h at 25 °C.

#### 3.2.5. Biocatalytic Reaction to Reduce 3-Quinuclidinone to (*R*)-(−)-3-Quinuclidinol with a Coenzyme Regenerating System

The 3-quinuclidinone conversion reaction was carried out with the coenzyme regeneration system by LSADH. The reaction mixture consisted of 200 mM KPB (pH 7.0), 5% (*w*/*v*) 3-quinuclidinone, 1 mM NAD^+^, various *E. coli* biocatalysts and 5% (*v*/*v*) 2-propanol in a total volume of 1 mL. The reaction proceeded in an unsealed 5 mL vial to accelerate vaporization of the acetone produced at 25 °C with shaking (2500 rpm, Taitec, Saitama, Japan) for 24 h. Both 3-quinuclidinone and 2-propanol were added to the reaction mixture at 8 h intervals resulting in 10% or 15% 3-quinuclidinone/2-propanol in the reaction mixture. The *E. coli* biocatalysts used in Table 2 were as follows: (a) *E. coli* BL21 (DE3)/pET28-QNR and *E. coli* BL21/pKELA (total 5 mL or 10 mL culture broth), (b) *E. coli* BL21(DE3) pET28-bacC and *E. coli* BL21/pKELA (total 5 mL or 10 mL culture broth), (c) *E. coli* BL21 (DE3)/pETDuet-QNR/pRSFDuet-LSADH (total 5 mL culture broth), (d) *E. coli* BL21 (DE3)/pET28-bacC-LSADH) (total 5 mL culture broth), (e) immobilized *E. coli* BL21 (DE3)/pET28-QNR and *E. coli* BL21/pKELA (each prepared from 5 mL culture broth), and (f) immobilized *E. coli* BL21(DE3)/pET28-bacC and *E. coli* BL21/pKELA (each prepared from 5 mL culture broth). After the reaction, 1 mL of the reaction mixture was centrifuged (18,000*g*, 5 min) and mixed with 6 N NaOH (0.2 mL), and the product was extracted twice with 1-butanol (0.5 mL), which contained 5 mM 1-octanol as an internal standard. The 1-butanol layer was thoroughly dried with anhydrous Na_2_SO_4_ and analyzed by gas chromatography (GC) after centrifugation as described below.

#### 3.2.6. Product Analysis by GC

The enantiomer of 3-quinuclidinol was analyzed by a GC system (HP 6890, Hewlett Packard, CA, USA) equipped with a chiral capillary column (CP-cyclodextrin-β-236-N19, 0.25 mm × 25 m, Varian, CA, USA) with a flame ionization detector. The GC conditions were as follows: the column temperature program ramped from 70 °C to 180 °C at 10 °C min^−1^, the injection and detection temperatures were 250 °C, and the He flow rate was 3.3 mL min^−1^ with a linear velocity of 50 cm s^−1^ and a split ratio of 50. The retention times were 8.17 min for the 1-octanol internal standard, 10.94 min for 3-quinuclidinon, 12.69 min for (*S* )-(+)-3-quinuclidinol and 12.77 min for (*R*)-(−)-3-quinuclidinol.

## 4. Conclusions

We successfully constructed a number of *E. coli* biocatalysts, in which the QNR/bacC and LSADH genes were independently or dependently expressed, to produce optically pure (*R*)-(−)-3-quinuclidinol. By optimizing the reaction conditions including the combination of *E. coli* biocatalysts, a production level of approximately 100 mg/mL was attained with the intact *E. coli* biocatalyst process. Moreover, the combination of immobilized *E. coli*/pET28-QNR and *E. coli*/pKELA biocatalysts improved the production level to 150 mg/mL (939 mM) with 100% conversion.

## Figures and Tables

**Table 1:** Enzyme activity of recombinant *E. coli* biocatalyst.

Plasmid for expression	3-Quinuclidionone reduction (U/mL culture broth)	2-Propanol oxidation (U/mL culture broth)
pET28-QNR *	8.4	0
pET28-bacC *	0.3	0
pETDuet-QNR/pRSFDuet-LSADH	0.3	0.12
pET28-bacC-LSADH	0.1	0.09
pKELA	0	1.0

* The specific activity of QNR was 12.2 U/mg protein, and that of bacC was 0.9 U/mg protein.

**Table 2:** Production of (*R*)-(−)-3-quinuclidinol by various combinations of biocatalysts.

	Biocatalyst	Amount of cells a	Cells-mixing ratio	Production level b, molar conversion
	Resting cells			
(a)	pET28-QNR, pKELA	40.6 mg	4:1	100 mg/mL ± 1, 100%
		42.3 mg	1:1	100 mg/mL ± 1, 100%
		43.9 mg	1:4	96 mg/mL ± 2, 96%
		42.3 mg	1:1	51 mg/mL ± 3, 34% c
		84.5 mg	1:1	63 mg/mL ± 3, 42%^c^
(b)	pET28-bacC, pKELA	40.2 mg	4:1	98 mg/mL ± 2, 98%
		42.0 mg	1:1	99 mg/mL ± 1, 99%
		43.8 mg	1:4	65 mg/mL ± 3, 65%
		42.0 mg	1:1	34 mg/mL ± 3, 22% c
		84.0 mg	1:1	58 mg/mL ± 4, 39%^c^
(c)	pETDuet-QNR/pRSFDuet-LSADH	35.5 mg	-	100 mg/mL ± 1, 100%
(d)	pET28-bacC-LSADH	36.5 mg	-	98 mg/mL ± 2, 98%

	Immobilized cells			
(e)	pET28-QNR, pKELA	84.5 mg	1:1	150 mg/mL ± 2, 100% c
(f)	pET28-bacC, pKELA	84.0 mg	1:1	24 mg/mL ± 3, 16% b

a Total amount (wet weight) of recombinant *E. coli* cells was adjusted by harvesting from a constant culture broth (5 mL or 10 mL) with varying amounts of each of the recombinant *E. coli* cells;

b The average of three independent conversion experiments with standard deviation in shown;

c The final concentration of 3-quinuclidinone was 15% (*w*/*v*) and the reaction time was 48 h.
